# Purification of Antilisterial Peptide (Subtilosin A) from Novel *Bacillus tequilensis* FR9 and Demonstrate Their Pathogen Invasion Protection Ability Using Human Carcinoma Cell Line

**DOI:** 10.3389/fmicb.2016.01910

**Published:** 2016-12-01

**Authors:** Rizwana Parveen Rani, Marimuthu Anandharaj, Subramani Hema, Ramasamy Deepika, Abraham David Ravindran

**Affiliations:** ^1^Department of Biology, The Gandhigram Rural Institute – Deemed UniversityGandhigram, India; ^2^Biodiversity Research Center, Academia SinicaTaipei, Taiwan

**Keywords:** probiotic, *Bacillus tequilensis*, bacteriocin, Subtilosin A, adhesion assay, cholesterol reduction

## Abstract

This study focuses on isolation, screening, and characterization of novel probiotics from gastrointestinal tract of free-range chicken (*Gallus gallus domesticus*). Fifty seven colonies were isolated and three isolates (FR4, FR9, and FR12) were selected and identified as *Lactobacillus gasseri* FR4, *Bacillus tequilensis* FR9, and *L. animalis* FR12 by 16S rRNA sequencing. Three strains were able to survive in stimulated acidic and bile conditions and inhibit the growth of pathogens. Especially, FR9 exhibited maximum inhibition against *Listeria monocytogenes* and none of them exhibited hemolytic activity. Native-PAGE revealed the presence of low molecular weight (3.4–5.0 KDa) antimicrobial peptide. The peptide was further purified by Sephadex G-50 column and RP-HPLC using C18 column. N-terminal amino acid sequencing of antimicrobial peptide showed 100% consensus to antilisterial peptide Subtilosin A and SboA gene was amplified from FR9 genome. FR9 showed maximum aggregation activity, exopolysaccharide production (85.46 mg/L) and cholesterol assimilation (63.12 ± 0.05 μg/mL). Strong adhesion property (12.6%) and pathogen invasion protection ability was revealed by *B. tequilensis* FR9 towards HCT-116 human colon carcinoma cell line. This is the first study to demonstrate antilisterial Subtilosin A production of *B. tequilensis*. Our results indicate that *B. tequilensis* FR9 strain furnish the essential characteristics of a potential probiotics and might be incorporated into human and animal food supplements.

## Introduction

Probiotic microorganisms are live bacteria, which modulates the host immune system and maintain the intestinal microbial balance (Joint FAO/WHO, 2002). Majority of the probiotic bacteria belongs to the genus *Lactobacillus* and *Bifidobacterium*, and considered as “Generally Recognized As Safe” (GRAS) by the United States Food and Drug Administration (FDA; Rubio et al., 2014). However, recent researches demonstrated that several *Bacillus* species also furnish the essential probiotic characteristic and produce group of antimicrobial peptides with broader inhibition spectrum (Khochamit et al., 2015). Probiotic *Lactobacillus* and *Bacillus* strains isolated from diverse sources are used as probiotic candidates and it is unlikely that each species/strain possesses all the preferred characteristics that will make it a felicitous probiotic (Swain et al., 2014). A potential probiotic bacteria must fulfill certain fundamental criteria, such as ability to adhere on intestinal epithelium, to overcome potential obstruction, including low pH of the stomach, presence of bile acids in intestines, compete/antagonize enteric pathogens, susceptibility to commonly used antibiotics, cholesterol lowering effect and colonize the intestinal cell wall (Garriga et al., 1998; Chiang and Pan, 2012; Anandharaj et al., 2015). Probiotic microbes also synthesis various biologically active compounds including enzymes, bacteriocins, exopolysaccharides (EPS), vitamins, and organic acids and these compounds has immense industrial applications (Nel et al., 2001; Walling et al., 2005).

Contrast to the gram-positive lactic acid bacteria (LAB), *Bacillus* spp. produce diverse antimicrobial compounds (i.e., bacteriocins), exhibiting broader inhibition spectra against various food borne pathogens (Abriouel et al., 2011; Khochamit et al., 2015). Antimicrobial peptides from various *Bacillus* spp. such as *B. subtilis, B. megaterium, B. cereus, B. coagulans, B. thuringiensis* have been studied intensively, however, the bacteriocins of *B. tequilensis* has not yet been studied. In this work, we have demonstrated the Subtilosin A production by *B. tequilensis* FR9. Subtilosin A is an unique cyclic bacteriocin with three intra molecular bridges produced by *B. tequilensis* and expressed by *sboA-albABCDEFG* gene cluster, which is responsible for the antibacterial activity against the common food borne pathogen *Listeria monocytogenes* (Huang et al., 2009; Fluhe et al., 2012; Khochamit et al., 2015). Moreover, several probiotics have been reported for their EPS production which enhances probiotic colonization by cell-cell interaction in gastrointestinal tract (GIT; Kanmani et al., 2013) and exhibits antimicrobial, immunostimulatory, antioxidant, and antitumor activities (Pan and Mei, 2010; Zhang et al., 2013; Ibarburu et al., 2015). Abundance of EPS around the cells greatly influences bacterial aggregation (Wang et al., 2014; Dertli et al., 2015), cell surface hydrophobicity (Collado et al., 2008), biofilm formation, thus competitively excludes adhesion of pathogens on intestinal cell lines (Collado et al., 2008; Walter et al., 2008). Previously, pathogen invasion protective ability of various LAB strains were demonstrated against *Salmonella typhimurium* (Golowczyc et al., 2011), *Klebsiella pneumonia*, and *Pseudomonas aeruginosa* (Khan and Kang, 2016).

Free-range chickens are mainly fed household food/conventional chicken feeds devoid of antibiotics making them susceptible to a variety of infections which may be controlled by endogenous probiotic microbial communities. The selection and screening processes for strain isolation from broiler chickens have been extensively studied, whereas characterization of probiotic microorganisms isolated from free-range chicken is scarce (Garriga et al., 1998; Musikasang et al., 2009; Taheri et al., 2009). Hence, in this study we intend to isolate the potential probiotic bacteria from GIT of free-range chicken (*Gallus gallus domesticus*) and investigate their probiotic potential and antimicrobial activity against several food borne pathogens. Further, we have also purified the antimicrobial peptide and demonstrated their pathogen invasion protection ability against *L. monocytogenes* and *Enterococcus faecalis* to verify its intestinal barrier function.

## Materials and Methods

### Ethical Statement

Free-range Chickens used in this study were purchased from local farmers. Since the free-range chickens are wild animals, the ethical review process is not needed for this study. However, the chickens were sacrificed according to the guidelines of Committee for the Purpose of Control and Supervision of Experimentation on Animals (CPCSEA), India.

### Bacterial Isolation and Identification

Five healthy, 20-days old indigenous free-range chickens were sacrificed by cervical dislocation. Contents of GIT (i.e., crop, gizzard, small intestine, large intestine, and caeca) were washed with 70% ethanol followed by sterile H_2_O. The contents were plated on De Man Rogosa and Sharpe (MRS) agar (HiMedia, India), supplemented with 0.02% bromocresol purple and incubated at 37°C for 36 h. Totally, 57 colonies were isolated from the higher dilution plates and 12 isolates displayed clear halo zone around the colonies confirming the lactic acid production. To further scrutinize the selected isolates, we performed acid and bile tolerance assay. Among 12 isolates, FR4, FR9, and FR12 exhibited more resistance to tested pH, bile concentration and was used for further study. FR4 and FR12 isolates were identified as *Lactobacillus* spp. and FR9 isolate was identified as *Bacillus* sp.. To identify the species level, 16S rRNA sequencing was performed. Obtained sequences were assembled using BioEdit software (7.00) and identified using nucleotide Basic Local Alignment Search Tool (BLASTn). Phylogenetic relationship was confirmed by neighbor-joining phylogenetic tree.

### Evaluation of Probiotic Properties under Stimulated Conditions

#### Acid Tolerance

Tolerance to acidic conditions was examined by inoculating overnight grown bacterial cells in pH adjusted MRS broth (1.0, 2.0, 3.0, and control 7.0) and incubated for 3 h at 37 ± 2°C. Viable cell count was determined by plating on MRS agar and incubated for 24 h at 37 ± 2°C. The cell count was expressed as log value of colony-forming units per mL (log CFU/mL). Survival percentage was calculated as follows: % survival = final (log CFU/mL)/control (log CFU/mL) × 100.

#### Bile Tolerance

Bile tolerance ability of bacterial strains was determined by using MRS broth supplemented with (0.3 or 0.5% w/v) bile salts (Oxgall, Merck) and MRS without bile salts served as control. Overnight grown bacterial cells (A_600_ = 1.5) were harvested, washed with distilled H_2_O, resuspended in MRS broth and incubated at 37 ± 2°C. One mL sample was withdrawn after 3 and 5 h, subsequently plated onto MRS agar. Bacterial viability was assessed as mentioned in the previous experiment.

#### Antibiotic Resistance Profile

Antibiotic susceptibility was examined using the method recommended by Clinical and Laboratory Standards Institute (CLSI). Eleven different antibiotic disks (HiMedia, India) such as, Ampicillin (10 μg), Amoxicillin (5 μg), Cefuroxime (30 μg), Chloramphenicol (30 μg), Erythromycin (20 μg), Kanamycin (30 μg), Gentamicin (10 μg), Metronidazole (20 μg), Penicillin (30 μg), Tetracycline (20 μg), and Novobiocin (30 μg) were used. MRS agar plates were seeded with bacterial strains and incubated according to National committee for clinical laboratory standards (NCCLS) guidelines for Kirby Bauer test (NCCLS, 1997). Breakpoints for the interpretation of inhibition zone were expressed in terms of resistance (++), Intermediate susceptible (+), and susceptible (-) as described by CLSI (2011).

#### Antimicrobial Activity

Antimicrobial activity was performed using agar well-diffusion method (Anandharaj et al., 2015). Cell-free culture supernatants (CFCS) were collected by centrifugation (10,000 × *g*, 4°C, 20 min) and filtered through 0.22 μm membrane filter (Millipore, USA). To demonstrate the antimicrobial activity, 10 μL test pathogens (10^7^–10^9^ CFU/mL) were spread onto agar plates, 100 μL pH neutralized CFCS (pH 6.5) was added to each well and incubated (Supplementary Table S1). Antimicrobial activity was expressed as Arbitrary unit (AU) using following formula (Abbasiliasi et al., 2014).

$$\mathrm{Antimicrobial}\;\mathrm{activity}(\mathrm{AU})(\frac{mm^{2}}{mL})=\frac{L_{Z}-L_{S}}{V}$$

Where, *Lz* = clear zone area (*mm*^2^), *Ls* = well area (*mm*^2^), *V* = volume of sample (*mL*).

### Purification of Bacteriocin

#### Partial Purification

Partial purification of bacteriocin (PPB) was performed according to Khochamit et al. (2015). Bacteriocin was precipitated with 40% (w/v) ammonium sulfate (AS), collected by centrifugation (10000 × *g* for 20 min, 4°C) and dissolved in phosphate buffered saline (PBS). Dialysis was performed against same buffer using 1 KDa cutoff membrane (16 h, 4°C). Protein concentration was measured by Bradford method (Protein Assay Kit, BioRad) and antagonistic activity was determined.

#### Purification

Partial purification of bacteriocin was purified using Sephadex G-50 (Sigma, USA) column connected with AKTA prime plus (GE healthcare, Uppsala, Sweden) protein purification system equilibrated with 10 mM Phosphate buffer (pH 7). Three milliliter PPB was loaded on to pre-equilibrated column and elution was performed at the flow rate of 1 mL/min. The elution was observed at 280 nm using UV detector. Fractions showed peaks were collected (3 mL) and antimicrobial activity was analyzed. Active fractions were pooled together and further purified using Reverse-phase (RP) HPLC coupled with C18 column. Briefly, 20 μL of purified fractions were injected into the column and eluted at the flow rate of 1 mL/min using solvent A (60% methanol) and solvent B (40% water) for 45 min. Protein concentration was monitored at 220 and 284 nm. Fractions were collected manually and antimicrobial activity was assessed.

### Characterization of Bacteriocin

#### SDS-PAGE and Native-PAGE

The molecular weight mass of purified bacteriocin was determined by SDS-PAGE (5% stacking and 12.5% resolving) stained with coomassie brilliant blue R250. Native-PAGE (5% stacking and 12.5% resolving) was performed without heating the protein. Subsequently, the gel was overlaid on nutrient agar plate coated with *L. monocytogenes* and incubated for 24 h at 37 ± 2°C.

#### Amino Acid Sequencing

To determine the amino acid sequence of bacteriocin, single band with antimicrobial activity from SDS-PAGE was transferred to polyvinylidene fluoride (PVDF) membrane and excised for N-terminal sequencing using Edman degradation method. The resulted sequence was identified using protein BLAST.

#### Fourier Transform Infra-Red (FTIR) Analysis

Surface functional groups of purified BLIS were unraveled by Perkin-Elmer infrared spectrophotometer (India). Purified BLIS was mixed with KBr (spectroscopic grade) and pellet was prepared with the size of about 10–13 mm diameter and 1 mm thickness. Sample was scanned in transmission mode with a resolution of 4 cm^-1^ at 4000–400 cm^-1^ range and functional groups were compared with previously published literature (Coates, 2000; Ajesh et al., 2013).

#### Molecular Identification of Bacteriocin Gene

To identify the specific bacteriocin gene, primers were designed (**Table 1**) for five putative *Bacillus* sp. antimicrobial genes (cerein, Subtilosin A, thuricin H, lichenicidin, and ericins) and was amplified from FR9 genomic DNA.

**Table 1:** Gene specific primers used for the amplification of bacteriocin genes in this study.

Bacteriocin gene	Bacterial source	Sequence (5′ to 3′)	Expected amplicon size (bp)	Reference
Cerein	*Bacillus cereus*	Cer7B-F:CCCTCTATATGAGGGAGTAA	416	This study
		Cer7B-R: GTTTAATAATCTATACAGTA		
Subtilosin A	*Bacillus subtilis*	SboA-F: CATATGAAAAAAGCTGTCATTG	394	This study
		SboA-R: AAGCTTTTACCCCCATAGACC		
Thuricin H	*Bacillus thuringiensis*	Thu17-F: AGTATGTGCAGCATGTTCTG	555	This study
		Thu17-R: ATAAACACTCTCACATTTTT		
Lichenicidin	*Bacillus licheniformis*	LanA2-F: ATGTCAAAAAAGGAAATGAT	225	This study
		LanA2-R: TTAGTTACAGCTTGGCATGC		
Ericins	*Bacillus subtilis*	EriSa-F: GTGACTAATATGTCAAAGTT	171	This study
		EriSa-R: TCAGCACTTAGCAAATGTTG		
albA	*Bacillus tequilensis*	albA-F:CTAAATAAGCTGGACCACGTCTT	1347	This study
		albA-R: TTGTTTATAGAGCAGATGTTTCC		

### Evaluation of Safety and Functional Properties

#### Hemolytic and β-Glucosidase Activity

Probiotic strains were streaked on nutrient agar plates supplemented with 5% (w/v) sterile defibrinated sheep blood and incubated for 48 h at 37 ± 2°C. Hemolytic activity was observed post partial hydrolysis and formation of green hued zones (α hemolysis), clear zone of hydrolysis (β hemolysis), or no zone around the colonies (γ hemolysis). The β-glucosidase activity was determined using *p*-nitrophenyl-D-glucose (pNPG) as substrate. Briefly, the culture supernatant was mixed with 50 mM pNPG, 50 mM citrate buffer, 15 mM CaCl_2_, and incubated at 40°C for 10 min. Absorbance was measured at 410 nm and *p*-nitrophenol release was calculated using standards.

### Bacterial Aggregation Activity

#### Autoaggregation

Autoaggregation was performed as described in Collado et al. (2008). Briefly, 10^7^ cells/mL of probiotic cells were harvested, washed with PBS (pH 7.2) and resuspended in same buffer. Consecutively, bacterial suspensions were incubated at 37 ± 2°C and monitored at different time intervals (0, 1, 2, 3, 4, and 5 h). The percentage of autoaggregation was expressed as: A% = (*A*_0_-*A*_t_)/*A*_0_^∗^100

Where, *A*_0_ represents the absorbance (*A*_600_ nm) at 0 h and *A*_t_ represents the absorbance at different time intervals.

#### Coaggregation

Coaggregation was demonstrated as described in Collado et al. (2008). Probiotic cell suspensions were prepared as illustrated in autoaggregation analysis. Equal volumes (500 μL) of various probiotic and pathogenic (*L. monocytogenes, Escherichia coli*, and *Enterococcus faecalis*) cell suspensions were mixed and incubated at room temperature. Absorbance (*A*_600_ nm) of above mixtures as well as individual bacterial suspensions was monitored during incubation. Coaggregation was calculated as:

$$[(A_{\mathrm{pat}}+A_{\mathrm{probio}})/2-(A_{\mathrm{mix}})/(A_{\mathrm{pat}}+A_{\mathrm{probio}})/2]*100$$

Where, *A*_pat_ and *A*_probio_ represents absorbance at *A*_600_ nm of individual bacterial suspensions in control tubes, *A*_mix_ represents the absorbance of mixed bacterial suspension at different time tested.

#### Microbial Adhesion to Hydrocarbons (MATH)

Microbial Adhesion to Hydrocarbons was determined as described in Collado et al. (2008). Probiotic cells were harvested, washed and resuspended in 3 mL of PBS. *A*_600_ was adjusted to 0.2–0.3 to obtain 10^7^ CFU/mL of bacteria (*A*_0_). One mL hydrocarbons (xylene, *n*-hexadecane and toluene) were added with cell suspension to form a two-phase system. After pre-incubation (10 min), the two-phase system was vortexed (2 min), incubated (20 min), and *A*_600_ of aqueous phase was measured (*A*_1_). Percentage of MATH was calculated according to the following equation.

$$\mathrm{MATH}\%=1-A_{1}/A_{0}$$

#### Screening for EPS Production

Exopolysaccharide production was examined by streaking the probiotic strains on MRS agar supplemented with 2 or 4% (w/v) glucose/lactose/sucrose. After incubation (24–48 h at 37 ± 2°C), plates were observed for the appearance of colonies exhibiting a mucoid feature. The production of biopolymer was confirmed by mixing a scrap of mucoid substance in 2 mL of absolute alcohol additionally by observing the mucoid substance in scanning electron microscopy (SEM).

#### Qualitative Determination of Bile Salt Hydrolase (BSH)

Bile salt deconjugation ability was studied as described by Kumar et al. (2012). Bacterial cells were plated on MRS-Thio agar fortified with 0.5% (w/v) taurodeoxycholate (TDC) and 0.37 g of CaCl_2_/L preceded by incubation at 30 ± 2°C for 24–48 h. MRS-Thio agar medium plates without TDC served as control.

#### Cholesterol Assimilation Assay

Water-soluble cholesterol (polyoxyethanyl-cholesteryl sebacate, Sigma, USA) was dissolved in 50% ethanol (5 mg/mL), filter sterilized and added to MRS-Thio broth supplemented with 0.3% ox-bile at a final concentration of 50–200 μg/mL. Medium was inoculated (1% v/v) with probiotic strains and incubated at 37 ± 2°C for 20 h. Subsequently, cells were harvested (10,000 × *g* at 48°C for 10 min) and remaining cholesterol concentration in broth was determined using the method of Rudel and Morris (1973). Cholesterol assimilated by probiotic strains was determined as follows:

Cholesterol assimilation(μg/mL)= (C1-C2)/(W2-W1)

Where, C1 and C2 represent the cholesterol concentration of the uninoculated and inoculated medium, respectively; W1 and W2 represent the weight of culture per milliliter of medium before and after the incubation period.

#### HCT-116 Cell Line Growth Condition

Adhesion assay and pathogen invasion protection by *B. tequilensis* FR9 was demonstrated using human colon carcinoma cell line HCT-116. The HCT-116 cells were cultured in Roswell Park Memorial Institute (RPMI)1640 medium supplemented with 25 mM HEPES buffer, 25 mM sodium bicarbonate, 300 mM L-glutamate, and 10% heat inactivated fetal bovine serum at 37°C in 5% CO_2_ and 95% air. Cells were passaged every 2 days and experiments were conducted after 20–25 passages of cell line (undifferentiated state).

#### Adhesion Assay on HCT-116 Cell Line

*In vitro* adhesion of *B. tequilensis* FR9 on HCT-116 human colon carcinoma epithelial cells was conducted as described by Das and Goyal (2014) with some modifications. To obtain HCT-116 monolayers, each well of the tissue culture plates (six well) were seeded with 4 × 10^4^ cells per cm^2^ and incubated at 37°C for 24 h. RPMI-1640 medium was aspirated out after reaching 80% confluency, and cells were washed with PBS (pH 7.4) buffer. Simultaneously, overnight grown *B. tequilensis* FR9 cells were harvested by centrifugation, washed with PBS buffer and resuspended in RPMI-1640 medium (devoid of antibiotic and serum) at 1 × 10^8^ CFU/mL concentration. To perform the adhesion assay, 1 mL bacterial suspension was added to HCT-116 monolayers and incubated in humidified CO_2_ incubator (5% CO_2_) for 4 h at 37°C. Non-adhered cells were removed by PBS buffer wash (five times). To determine the percentage of adhesion, cells were lysed by sterile distilled water and plated on nutrient agar. Adhesion rate was calculated using the ratio between number of inoculated cells and number of colonies observed on nutrient agar plates. For the microscopic observation, HCT-116 cells along with adhered probiotic bacteria were fixed with 3 mL methanol and incubated at room temperature for 10 min. Methanol was removed completely and cells were stained with 0.1 % crystal violet (10 min), washed with ethanol to remove excess stain and examined under light microscope using bright field (BF) and Differential interference contrast (DIC) with 100× magnification (oil immersion).

#### Pathogen Invasion Protection Assay

The pathogen invasion protection ability of FR9 strain was demonstrated using human colon cancer cell line HCT-116 according to Khan and Kang (2016) and Golowczyc et al. (2011). HCT-116 cells were seeded in cell culture plates (4 × 10^4^ cell per cm^2^) to form monolayer. Then the monolayer of HCT-116 (post-confluence stage) was washed with sterile PBS (pH 7.4) and pre-incubated with 0.5 mL of well grown *B. tequilensis* FR9 (2 × 10^8^ CFU/mL in PBS) for 1 h at 37 ± 2°C in a 5% CO_2_ to 95% air atmosphere. After the incubation, monolayer along with probiotic microbes was washed twice with sterile PBS to remove the unbound microbes. Subsequently, 0.5 mL (2 × 10^8^ CFU/mL in PBS) bacterial pathogens (*L. monocytogenes* MTCC 657 and *E. faecalis* MTCC 439) as well as 0.5 mL RPMI-1640 media (Gibco, USA) were added to the plate and incubated for 1 h at 37 ± 2°C in a 5% CO_2_ to 95% air atmosphere. HCT-116 monolayer along with probiotics and test pathogens were washed twice with PBS and lysed by sterile distilled water. Finally, CFU/mL of invaded pathogen was counted on nutrient agar plates.

#### Scanning Electron Microscopic (SEM) Analysis

Scanning Electron Microscopic was performed to identify the EPS production as well as binding of cholesterol onto the bacterial cell surface. EPS producing cells or cholesterol treated cells were harvested by centrifugation (12,000 × *g* for 15 min at 4°C), resuspended in PBS buffer (pH 7.0), and air dried at 25°C to remove the moisture. Then the dried bacterial cells were mounted on the gold-coated SEM specimen stub, subsequently observed under the SEM (VEGA 3.0 TE Scan, USA).

### Statistical Analysis

All experiments were carried out in triplicates and the mean ± standard deviations (SD) values are represented in tables and figures. Significant differences between the samples were calculated by one way ANOVA with significant level *P* < 0.05. SPSS 13.0 (SPSS Inc., Chicago, IL, USA) and OriginPro 9.0 (MicroCal Software, Northampton, MA, USA) software’s were used for data analysis and graphical representation, respectively.

## Results and Discussion

### Screening and Identification of Bacterial Isolates

Totally, 57 isolates (i.e., 11-crop, 8-gizzard, 7-small intestine, 13-large intestine, and 18-caeca) were isolated from the higher dilutions plates. Among them, 12 isolates exhibited clear yellow halo zone around colonies on MRS-bromocresol purple plates. The selected 12 isolates were tested for their basic probiotic characteristics, such as acid and bile tolerance. The isolates FR4, FR9, and FR12 demonstrated higher resistance to the challenged acidic and bile conditions, hence selected for further characterization (Supplementary Table S2). FR4 and FR12 strains were identified as *Lactobacillus* spp. and FR9 was identified as *Bacillus* sp. (Hammes and Vogel, 1995). *Lactobacillus* strains were gram-positive rods, catalase negative and non-spore forming. *Bacillus* strain was gram-positive, spore forming rod, and catalase positive (Supplementary Figures S1 and S2). Results of morphological, biochemical, and fermentative characterizations are represented in Supplementary Tables S2 and S3.

Bacterial strains were identified by 16S rRNA sequencing and BLASTn analysis was performed. *Lactobacillus* FR4 revealed 100% identity to *Lactobacillus gasseri.* Similarly, *Lactobacillus* FR12 revealed 99% identity to *Lactobacillus animalis*, while *Bacillus* FR9 revealed 99% identity to *Bacillus tequilensis.* These three 16S rRNA gene sequences were deposited at NCBI GenBank under accession numbers KU587452, KU587453, and KU587454, respectively. Phylogenetic relationships are represented in Supplementary Figure S3. This is the first report to isolate and identify *B. tequilensis* FR9 from GIT of free-range chicken.

### Tolerance to Acidic pH

Acid tolerance is generally considered as an essential assessment criterion for probiotic evaluation, since the strains have to survive the acidic conditions of stomach and small intestine (Anandharaj et al., 2015). The viable cell counts were found to be <1.5 log CFU/mL after 3 h of exposure to pH 3, signifying their resistance and exhibiting survival of 82.22% (*L. gasseri* FR4), 82.86% (*B. tequilensis* FR9), and 80.91% (*L. animalis* FR12; **Table 2**). Among them, *B. tequilensis* FR9 exhibited more resistance to all tested acidic levels after 3 h. Similar kind of acid tolerance was observed in various *Lactobacillus* and *Bacillus* strains (Ahn et al., 2002; Beasley et al., 2004; Messaoudi et al., 2012; Khochamit et al., 2015; Nguyen et al., 2015).

**Table 2:** Effect of pH on the viability of FR4, FR9, and FR12 strains, incubated at various pH range (7, 1, 2, and 3), values are expressed as log CFU/mL, survival percentage and regression coefficient.

Strains	Control^a^ (log CFU/mL)	pH 1.0 (log CFU/mL)	SR^b^ (%)	pH 2.0 (log CFU/mL)	SR (%)	pH 3.0 (log CFU/mL)	SR (%)	Multiple R
FR4	6.47	1.59	24.57	3.71	57.34	5.32	82.22	0.857
FR9	6.83	2.38	34.85	4.86	71.15	5.66	82.86	0.904
FR12	7.86	1.68	21.37	3.46	44.02	6.36	80.91	0.826

*^a^Control: Bacterial cells were grown in MRS broth with pH 7.0.*

*^b^SR (Survival ratio in %) was calculated by cell numbers in MRS broth (pH 1.0–3.0)/cell numbers in control broth (pH 7.0) × 100.*

### Tolerance to Bile salts

Tolerance to bile salts is considered as a vital characteristic for colonization, metabolic activity of the strains in host gut, maintaining the equilibrium of gut microflora as well as lowering the host’s serum cholesterol (Anandharaj et al., 2014). The mean intestinal bile concentration is around 0.3% (w/v; Prasad et al., 1998), hence we challenged our probiotic strains with 0.3 and 0.5% bile concentration. All of the strains showed varying degrees of resistance to bile salts after 5 h exposure (**Table 3**). *L. gasseri* FR4, *B. tequilensis* FR9, and *L. animalis* FR12 strains were highly tolerant to 0.3% bile and exhibited slight reduction at 0.5% bile. In comparison, FR4 was found to be the least bile tolerant while FR9 was the most resistant strain. Our results are in agreement with the previously published works, in which the *Lactobacillus* and *Bacillus* strains retained their viability even after 5 h exposure to 0.3–0.5% bile concentration (Ahn et al., 2002; Messaoudi et al., 2012; Anandharaj et al., 2015).

**Table 3:** Effect of bile salt on the viability of FR4, FR9 and FR12 strains, incubated at various bile salt concentrations (0.3% to 0.5%).

Strains	3 h						5 h					
	Control^a^ (log CFU/mL)	0.3% bile salts (log CFU/mL)	SR^b^ (%)	0.5% bile salts (log CFU/mL)	SR (%)	Multiple R	Control^a^ (log CFU/mL)	0.3% bile salts (log CFU/mL)	SR^b^ (%)	0.5% bile salts (log CFU/mL)	SR (%)	Multiple R
FR4	6.53	5.32	81.47	4.26	65.23	0.758	7.78	5.78	74.29	4.34	55.78	0.864
FR9	8.06	6.86	85.11	5.93	73.57	0.903	8.61	6.46	75.02	5.93	68.87	0.911
FR12	7.76	5.76	74.22	4.90	63.14	0.812	8.43	5.63	66.78	4.56	54.09	0.791

*^a^Control: Bacterial cells were grown in MRS broth without supplementation of bile salt.*

*^b^SR (Survival ratio in %), was calculated by cell numbers in MRS (0.3 or 0.5% bile salts)/cell numbers in control (without bile salt) × 100.*

*Values are expressed as log CFU/mL, survival percentage and regression coefficient.*

### Antibiotic Resistance Profile

Bacteria predestined for probiotic use should be screened for antibiotic resistance to prevent any potential transfer of undesirable antibiotic resistance into the intestinal niche (Anandharaj and Sivasankari, 2014). Hence, probiotics strains were subjected to antibiotic susceptibility tests using eleven antibiotics. They were sensitive to 30 μg kanamycin and 30 μg novobiocin. However, FR4 and FR9 showed resistance to 50 μg amoxicillin, 30 μg chloramphenicol, and 10 μg gentamicin. *B. tequilensis* FR9 strain showed resistance to most of the antibiotics except kanamycin and novobiocin (Supplementary Table S4). Antibiotic resistance of these bacterial strains is considered intrinsic; hence it is non-transmissible (Argyri et al., 2013). Coetzee (2015) reported that *B. tequilensis* 5A2 was resistant to penicillin, tetracycline, streptomycin, and trimethoprim. Antibiotic resistance of *Lactobacillus* and *Bacillus* strains were also reported by other authors (Messaoudi et al., 2012; Anandharaj and Sivasankari, 2014; Nguyen et al., 2015).

### Determination of Antimicrobial Spectrum

Antagonist activity of probiotic bacteria facilitates the maintenance of gut microbiota balance and other physiological functions such as reduction of inflammatory bowel disease or colorectal cancer (Heilpern and Szilagyi, 2008). All three probiotic strains were able to inhibit growth of all Gram-positive, negative, and pathogenic yeast with varying extent (**Table 4**). Inhibition was not observed in *E. faecalis* by *L. animalis* FR12 whereas *B. tequilensis* FR9 strain showed broadest antimicrobial spectrum against all tested pathogens with the maximum inhibitory effect on *L. monocytogenes* (3250 AU/mL). Similar kind of results has been reported previously by various authors (Ahn et al., 2002; Strompfova and Laukova, 2007; Argyri et al., 2013; Anandharaj and Sivasankari, 2014; Khochamit et al., 2015). This is the first study reporting a broad spectrum antimicrobial activity of *B. tequilensis* FR9. Antagonism of *Lactobacillus* and *Bacillus* sp. is mainly due to the production of antimicrobial proteins and chemical compounds synthesized by secondary metabolic pathways (Anandharaj and Sivasankari, 2014).

**Table 4:** Antimicrobial activity of cell free culture supernatants (CFCS) of *L. gasseri* FR4, *B. tequilensis* FR9, and *L. animalis* FR12 strains against various pathogens (inhibition zone in mm ± standard deviation).

Bacterial Isolates	FR4	AU	FR9	AU	FR12	AU
*Pseudomonas aeruginosa* MTCC 741	9.50 ± 0.31^cd^	542.50	13.33 ± 0.13^e^	1416.88	11.81 ± 0.58^cd^	1034.76
*Escherichia coli* MTCC 2622	11.20 ± 0.13^e^	894.40	12.02 ± 0.42^e^	1084.80	10.09 ± 0.61^e^	658.08
*Klebsiella pneumoniae* MTCC 7028	10.90 ± 0.27^bc^	828.10	11.40 ± 0.12^b^	939.60	10.90 ± 0.28^b^	658.08
Diameter mean (Gram-negative)	10.53 ± 0.23	748.80	12.25 ± 0.22	1140.62	10.93 ± 0.49	834.64
*Bacillus cereus* MTCC 7278	7.36 ± 0.19^bc^	181.69	9.18 ± 0.18^b^	482.72	8.10 ± 0.07^b^	296.10
*Staphylococcus aureus* MTCC 3160	11.00 ± 0.81^bc^	850	13.20 ± 0.31^cd^	1382.40	9.00 ± 1.15^b^	450
*Listeria monocytogenes* MTCC 657	14.66 ± 0.67^c^	1789.15	19.00 ± 0.18^cd^	3250	15.09 ± 0.86^b^	1917
*Enterococcus faecalis* MTCC 439	6.33 ± 0.19^cd^	40.68	7.12 ± 0.24^e^	146.94	NS	0
Diameter mean (Gram-positive)	10.92 ± 0.37	832.46	12.59 ± 0.29	1225.08	8.04 ± 0.52	286.41
*Candida albicans* MTCC 3017	14.00 ± 0.29^f^	1600	17.01 ± 0.46^f^	2533.40	9.36 ± 0.64^f^	516
*Candida tropicalis* MTCC 184	12.00 ± 0.25^f^	1080	13.00 ± 0.51^f^	1330	11.00 ± 0.48^f^	850
*Lactobacillus oris* HMI68	1.07 ± 0.12^a^	125.80	6.98. ± 0.32^a^	127.20	6.63 ± 0.45^a^	79.56

*^1^Results were obtained by the neutralized supernatants of *L. gasseri* FR4, *B. tequilensis* FR9, and *L. animalis* FR12 strains at the stationary growth phase. ^2^Results are expressed as mean ± SD; *n* = 3. ^a-f^Means within a column with different lowercase letters are significantly different (*P* ≤ 0.05). NS, not significant (*P* > 0.05).*

### Antimicrobial Spectrum of FR9-PPB

Due to the hyper activity against *L. monocytogenes*, further characterization was performed. Antagonistic activity of FR9 PPB increased upto 1.5-fold compared to its CFCS, which indicates that the potency of bacteriocin was enhanced after partial purification (Supplementary Table S5). Percentage of difference in inhibition zone size was maximum (in terms of AU/ml) for *L. monocytogenes* (51.69%) and minimum for *E. faecalis* (10.73%).

### Purification of *B. tequilensis* FR9 Bacteriocin

**FIGURE 1:**
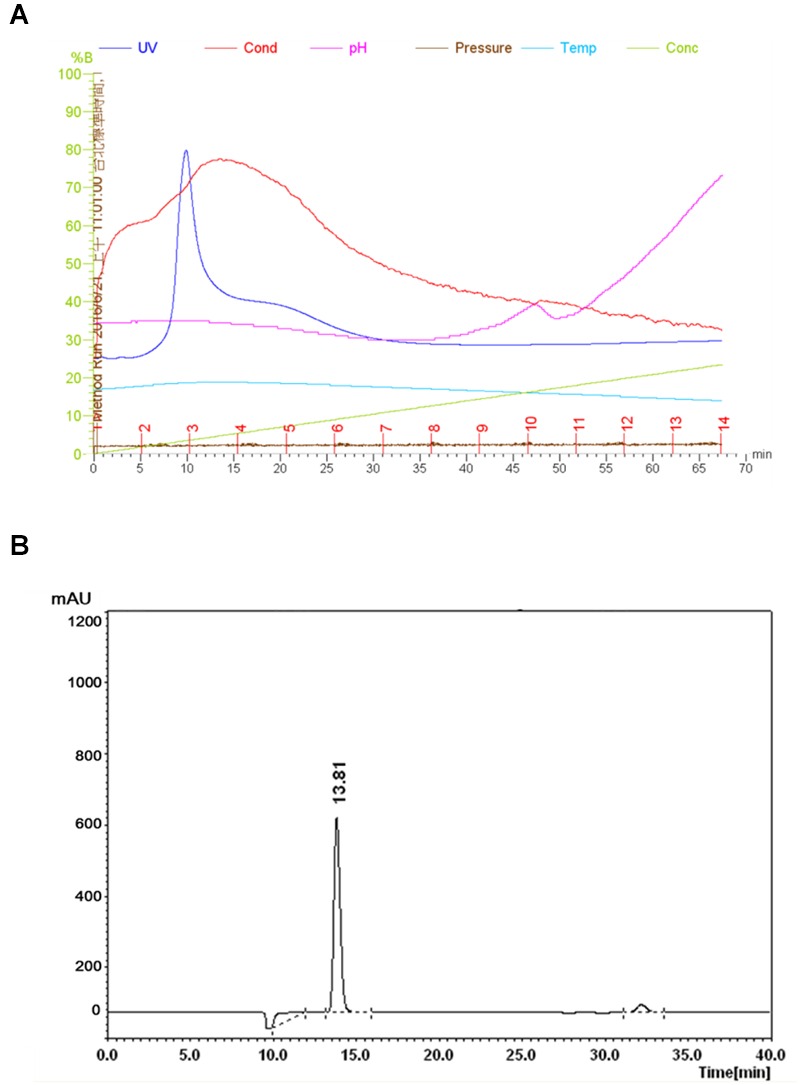
**Purification of Subtilosin A.** Sephadex G-50 chromatogram of Subtilosin A **(A)**. Reverse-phase (RP)-HPLC chromatogram of Subtilosin A **(B)**.

**FIGURE 2:**
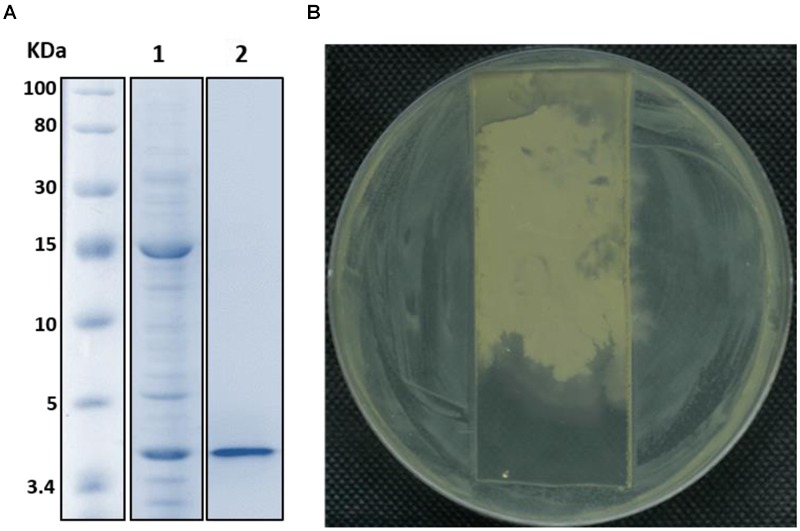
**SDS-PAGE (A)** and Native-PAGE **(B)** analysis of partially purified (Lane 1) and purified Subtilosin A (Lane 2).

**FIGURE 3:**
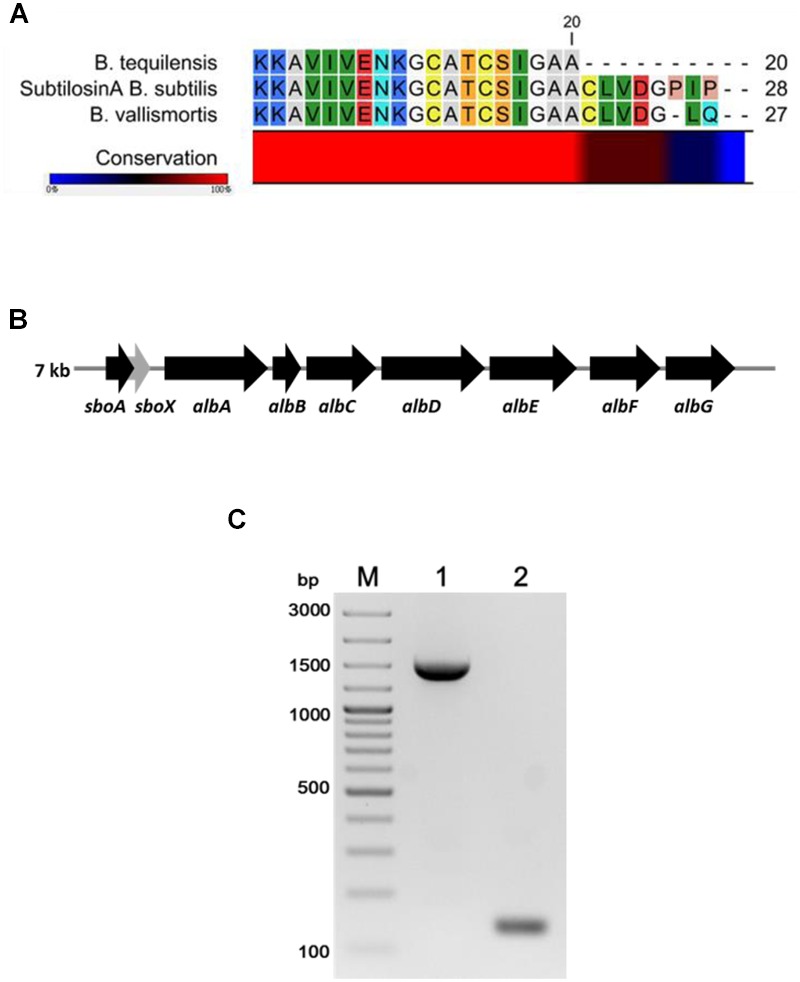
**Multiple sequence alignment of N-terminal amino acid sequence of *B. tequilensis* FR9 Subtilosin A with Sequence of *B. subtilis* and *B. vallismortis***(A)**.** Organization of structural genes in *SboAX-albABCDEFG* operon (7.0 kb) **(B)**. PCR amplification of albA (Lane 1) and SboA (Lane 2) from *B. tequilensis* FR9 genome **(C)**.

The FR9-PPB was purified using Sephadex G50 column, six fractions were collected (**Figure 1A**) and their antimicrobial activity was determined. The fractions F2 and F3 showed activity. They were pooled together and further purified using semi-preparative RP-HPLC. A single distinctive peak was observed at retention time 13.81 min (**Figure 1B**). Molecular weight of purified fraction was determined by SDS and Native-PAGE. SDS-PAGE of PPB showed major band around 4.3 KDa (Lane 1), but a single band was observed at 4.3 KDa in RP-HPLC purified fraction (Lane 2), indicating that the bacteriocin was a low molecular weight protein (**Figure 2A**). These results were further supported by overlaid native-PAGE, which showed an inhibition of *L. monocytogenes* growth in the range of around 3.4–5 KDa (**Figure 2B**). Amino acid sequencing results revealed 100% consensus to Subtilosin A of *B. subtilis* and *B. vallismortis* (**Figure 3A**). This is the first report to identify the Subtilosin A from *B. tequilensis*. Similarly, Khochamit et al. (2015) also purified the antilisterial peptide from *B. subtilis* KKU213 and identified as Subtilosin A. Zheng et al. (1999) demonstrated the antilisterial activity of Subtilosin A from *B. subtilis.* Moreover, bacteriocins of *Bacillus* spp. have been used as natural preservatives in food, as substitutes to conventional antimicrobials against human and animal diseases (Sass et al., 2008).

### Amplification of Subtilosin A Gene

Gene specific primers (**Table 1**) were designed to amplify the Subtilosin A gene (SboA) and subtilosin maturase gene (albA) from SboAX-albABCDEFG operon to confirm the presence of entire gene cluster in *B. tequilensis* FR9 genome (**Figures 3B,C**). The amplified sboA (132 bp) and albA (1347 bp) genes were confirmed by sequencing and multiple sequence alignment showed 100% similarity to Subtilosin A gene cluster of *B. subtilis*.

### FTIR Analysis of Subtilosin A

**FIGURE 4:**
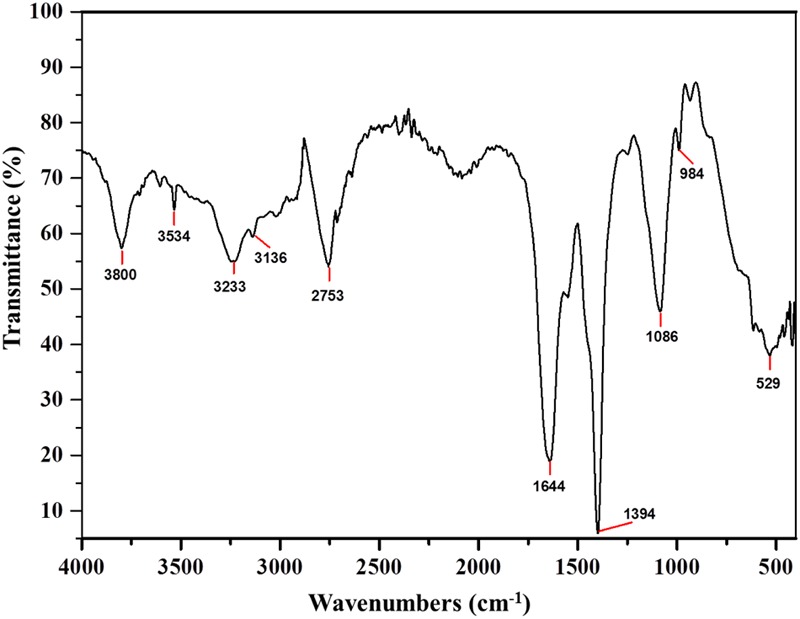
**Fourier Transform Infra-Red (FTIR) absorption spectra of Subtilosin A produced by *B. tequilensis* FR9**.

Fourier Transform Infra-Red is a reliable technique to identify the functional groups and thus unravel the putative mode of action of a bacteriocin (Motta et al., 2008). The FTIR spectra of Subtilosin A showed peaks at 1540, 1644, and 3233 cm^-1^ which confirms the existence of peptide bonds in the sample (**Figure 4**). The intense peak at 1644 cm^-1^ confirms the presence of primary amino group (amide I) with C = O stretch (Ajesh et al., 2013). Similarly, another intense peak at 1181 cm^-1^ revealed the presence of secondary amino group (amide II) having C-N stretch. Peaks at 2753 and 3534 cm^-1^ were due to the presence of N-CH3 (methylamino group) and dimeric OH stretch, respectively. Finally, the peak at 3136 cm^-1^ confirmed aromatic C-H stretch (Coates, 2000).

### Hemolytic Activity Assay

*In vitro* assessment of hemolytic activity for probiotics is one of the safety necessities used to assess potential probiotic strains (Joint FAO/WHO, 2002). All three strains exhibited γ hemolytic activity (i.e., no hemolysis) which further proves that these isolates are safe and reliable to use based on FDA, USA. Our results are supported by Luis-Villaseñor et al. (2011) in which *B. tequilensis* YC5-2 exhibited γ hemolytic activity and thus confirmed that, this isolate does not cause any health hazard.

### β-Glucosidase Activity

The β-galactosidase production is a common characteristic of several *Lactobacillus* and *Bacillus* species. In our study, all three strains produced β-galactosidase and *B. tequilensis* FR9 exhibited highest activity among others (7.65 ± 0.38 μmol/ml/min; **Table 5**). Our results are in agreement with the previous report of Palaniswamy and Govindaswamy (2016).

**Table 5:** β-glucosidase activity and exopolysaccharide (EPS) production by bacterial isolates.

Bacterial isolates	β-glucosidase activity (μmol/ml/min)	EPS production (mg/mL)
FR4	5.38 ± 0.26	64.32 ± 0.18
FR9	7.65 ± 0.38	85.46 ± 0.24
FR12	4.29 ± 0.17	43.49 ± 1.56

### Aggregation Activity

**FIGURE 5:**
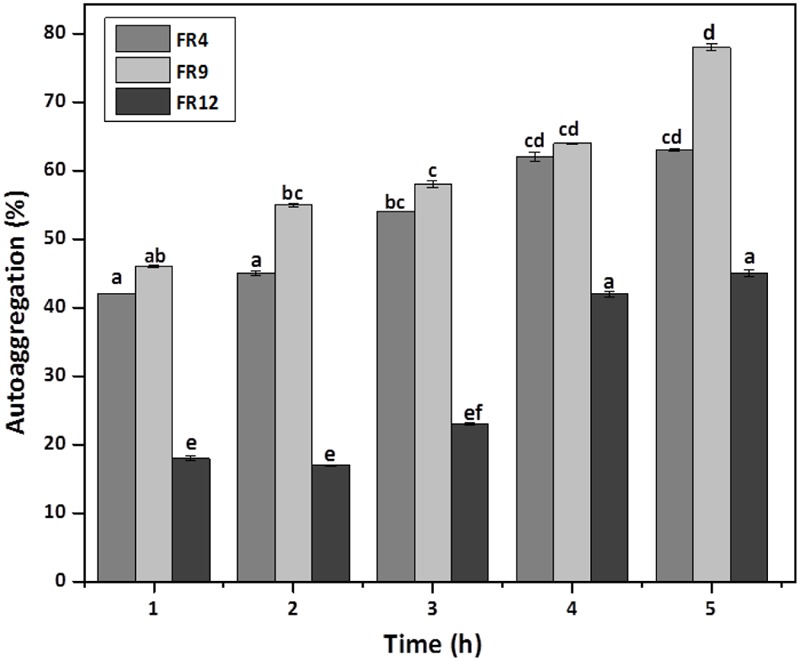
**Autoaggregation percentage of probiotic strains.** Values are expressed as mean ± SD (*n* = 3). The each bars with no common letters are significantly different (*P* < 0.05) according to the least significant difference (LSD) mean comparison test.

*In vitro* evaluation of autoaggregation and coaggregation ability with potential enteric pathogens could be used for primary screening and selection of best probiotic strains (Jankovic et al., 2012). Increased autoaggregation plays an important role in the adhesion of bacterial cells on intestinal epithelium and thus maintain the bacterial load in GIT (Del Re et al., 2000). Our results showed that all three strains displayed autoaggregation ability. Among them, *B. tequilensis* FR9 exhibited a strong autoaggregation (78%), followed by *L. gasseri* FR4 63% and *L. animalis* FR12 (45%; **Figure 5**). Similar effects were previously observed by various authors (Kos et al., 2003; Orłowski and Bielecka, 2006; Anandharaj et al., 2015).

**FIGURE 6:**
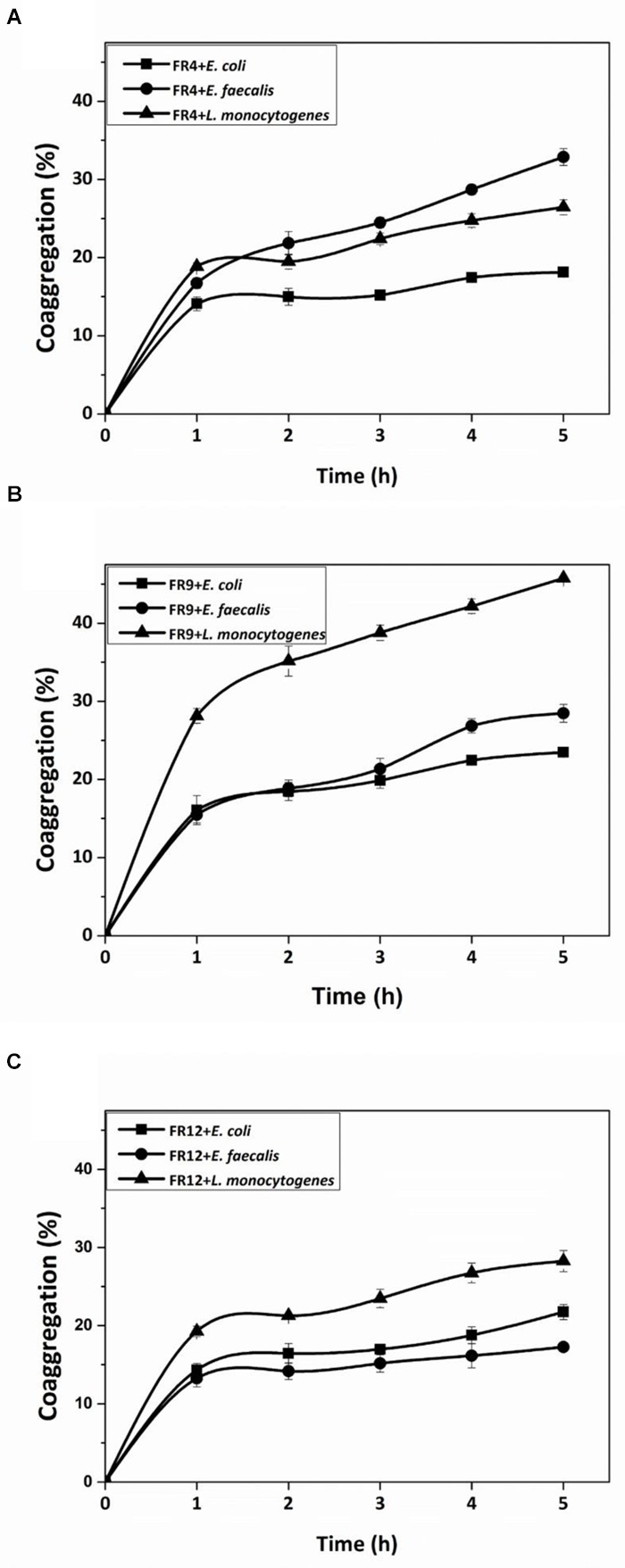
**Coaggregation percentages of *L. gasseri* FR4 (A)**, *B. tequilensis* FR9 **(B)**, and *L. animalis* FR12 **(C)** strains with three different pathogens (*E. coli, L. monocytogenes*, and *E. faecalis*). Values are expressed as mean ± SD (*n* = 3).

All three strains demonstrated coaggregation with *L. monocytogenes* ranging from 26 to 45% (**Figure 6**). *B. tequilensis* FR9 exhibited maximum coaggregation with *L. monocytogenes* (45%) followed by *L. animalis* FR12 (28%) while minimum coaggregation was recorded in *L. gasseri* FR4 strain (26%) after 5 h incubation. *Bacillus* sp. possessing ability to coaggregate with numerous pathogens is of unique interest with regard to its potential applications. Ability of *Bacillus* and *Lactobacillus* sp. to coaggregate with pathogens was observed by other authors (Collado et al., 2008; Anandharaj et al., 2015; Palaniswamy and Govindaswamy, 2016).

### Cell Surface Hydrophobicity

**FIGURE 7:**
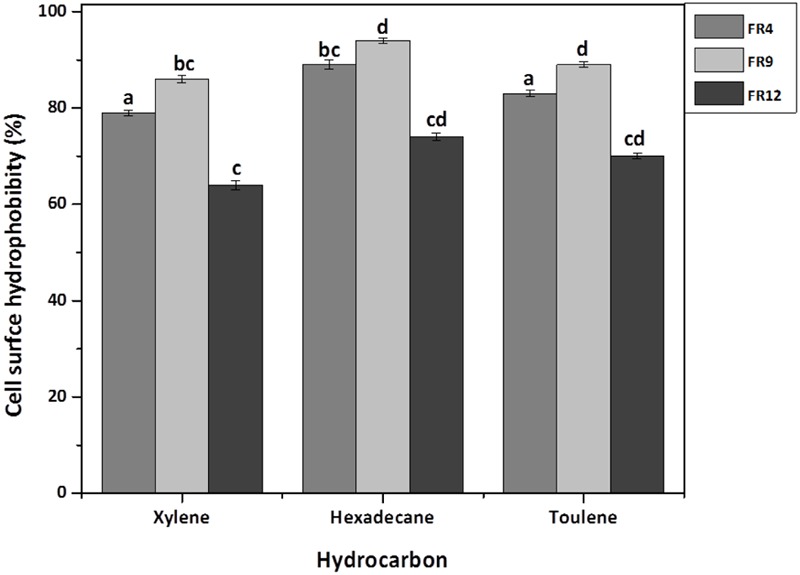
**Cell surface hydrophobicity [Microbial Adhesion to Hydrocarbons (MATH)] of probiotic using Xylene, Hexadecane, and Toluene.** Values are expressed as mean ± SD (*n* = 3). Each bars with no common letters are significantly different (*P* < 0.05) according to the LSD mean comparison test.

The high cell surface hydrophobicity of probiotic strains could indicate the ability to attach on intestinal epithelial cells thereby resist the digestive tract movement (Yu et al., 2007). Significant differences (*P* < 0.05) in hydrophobicity values were found among the probiotic strains. All strains showed greater hydrophobicity toward hexadecane and lowest toward xylene (**Figure 7**). Hydrophobicity for hexadecane was observed ranging from 74 to 94%. Similar hydrophobicity was observed by Torshizi et al. (2008) in *L. rhamnosus* (93.53%). The higher affinity toward hexadecane (apolar solvent) might be due to hydrophobic cell surface of the strains. Studies on physicochemistry of microbial cell surface has previously revealed that presence of glycoproteinaceous substance at the cell surface would lead to higher hydrophobicity (Kos et al., 2003).

### Screening for EPS Production

**FIGURE 8:**
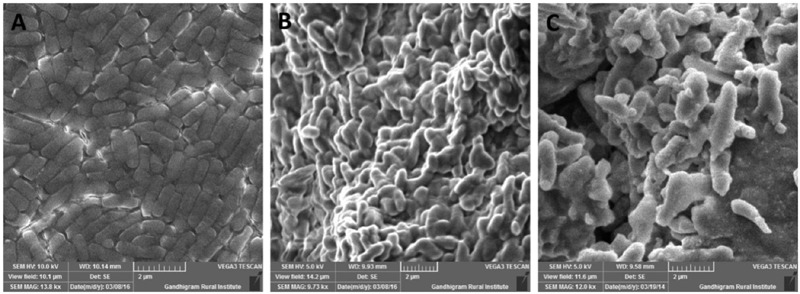
**Scanning electron microscopy (SEM) micrograph of *B. tequilensis* FR9 grown in (A)** MRS broth and **(B)** MRS broth with Sucrose for exopolysaccharide (EPS) production **(C)** MRS broth containing cholesterol fermented at 37°C for 20 h.

Exopolysaccharide is a major component present in cell surface of probiotic microorganisms. It has an extensive impact on their surface characteristics and acts as a protective barrier against harmful conditions (Anandharaj et al., 2015). In this study, *B. tequilensis* FR9 exhibited mucoid colony pattern in 4% sucrose supplemented MRS medium. Maximum EPS production was observed in *B. tequilensis* FR9 (85.46 mg/L; **Table 5**). **Figure 8B** provides strong evidence for the presence of EPS on FR9 cell surface. Our study reveals a positive correlation with the work done by Dertli et al. (2015), in which the cell surface of *L. johnsonii* FI9785 was covered by homo-polymeric EPS-1 and hetero-polymeric EPS-2. It has been reported that EPS may contribute to the aggregation properties as well as cell surface hydrophobicity of probiotic microorganism (Deepika et al., 2009).

### BSH Assay

Deconjugation of bile salts could prompt the reduction in serum cholesterol level (Begley et al., 2006). BSH activity was tested for all three selected strains by qualitative direct plate assay and all of them were able to hydrolyse TDC to precipitate deoxycholate on MRS-TDC agar. However, *B. tequilensis* FR9 was noted to display well defined silvery shine aspect.

### Cholesterol Assimilation Assay

**FIGURE 9:**
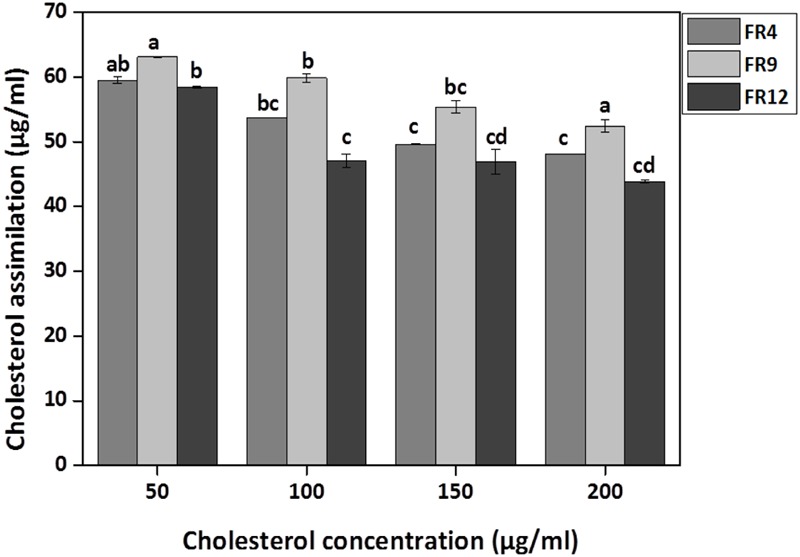
***In vitro* cholesterol assimilation by probiotic strains.** Values are expressed as mean ± SD (*n* = 3). Each bars with no common letters are significantly different (*P* < 0.05) according to the LSD mean comparison test.

All three strains exhibited higher cholesterol reduction ranging from 59.12 to 63.12 μg/mL in MRS broth. Among them, maximal (*P* ≤ 0.05) reduction was observed in *B. tequilensis* FR9 (63.12 μg/ml) whilst the minimum in *L. animalis* FR12 (59.12 μg/mL; **Figure 9**). Maximum cholesterol assimilation was observed with 50 μg/mL supplementation and thus illustrates that cholesterol concentration in media influences the cholesterol reduction. Through this study, it was observed that *B. tequilensis* FR9 has the innate ability to assimilate cholesterol which could play a pivotal role in controlling serum cholesterol levels. Furthermore, adherence of cholesterol on the surface of *B. tequilensis* FR9 was analyzed by SEM, which confirms the cholesterol assimilation mechanism (**Figures 8A,C**). This is the first study to report cholesterol assimilation of *B. tequilensis* FR9. Our results are in agreement with the work done by Lin and Chenn (2000) in which cholesterol-reducing abilities of *L. acidophilus* was investigated and found that hypocholesterolemic ability is due to the assimilation of cholesterol as well as its attachment to the surface. Anandharaj and Sivasankari (2014) reported that cholesterol assimilation by *L. oris* HMI68 ranged from 43.04 to 61.05 μg/mL after 24 h incubation. Similarly, Ramasamy et al. (2010) reported that *L. salivarius* I29 isolated from chicken intestine removed cholesterol (50.16%) from growth media (*P* < 0.005).

### Adhesion Assay

**FIGURE 10:**
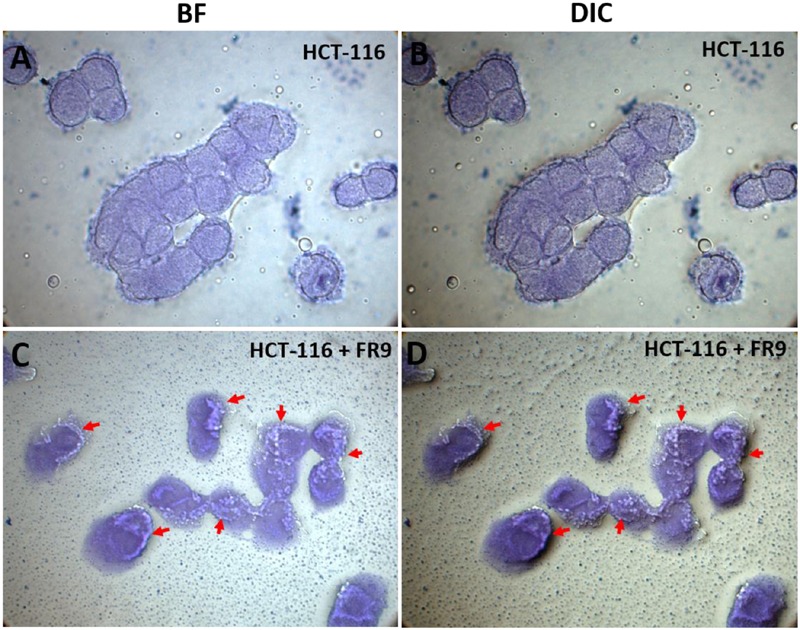
***In vitro* adhesion assay of *B. tequilensis* FR9 on HCT-116 colon carcinoma cell line observed under light microscope with 100× magnification (oil immersion).** The cells were stained with crystal violet. HCT-116 cell line alone observed under bright field (BF) **(A)** and differential interference contrast (DIC) **(B)**. HCT-116 cell line with *B. tequilensis* FR9 observed under BF **(C)** and DIC **(D)**. The arrows indicate the adhesion of FR9 cells on HCT-116 cell line.

Probiotic persistence and its colonization to digestive tract is the key necessity for bacteria to illustrate its advantageous effects for human wellbeing (Skrzypczak et al., 2015). In our study, HCT-116 colon carcinoma cell monolayer was used to demonstrate the adhesion properties of *B. tequilensis* FR9 both qualitatively and quantitatively. Qualitative examination was done by Crystal violet staining technique, thus provided visual confirmation of adhesion attributes (**Figure 10**). In BF and DIC micrographs (**Figures 10C,D**), bacterial adhesion on the HCT-116 surfaces was indicated with arrow marks. Subsequently, adhesion of *B. tequilensis* FR9 from initial inoculum was assessed by CFU cell counting. The relative percentage of adhesion was 12.6 ± 0.73% during post incubation. Hong et al. (2008) observed similar adhesion by *B. subtilis* strains on HT-29-16E, a mucus secreting colon carcinoma cell line. Similarly, Das and Goyal (2014) demonstrated a good adhesion percentage of 8.63 ± 3.03% by *L. plantarum* DM5 with HT-29 cell line. Adhesive property extensively relies on the origin, dosage, and is strain dependent. In our study, the *B. tequilensis* FR9 exhibited a good cell surface hydrophobicity of 86–94% and autoaggregation of 78%, which is an essential criterion for adhesion. These results support the adhesive capability of *B. tequilensis* FR9, and it may adhere on GIT and withstand the peristaltic movement of the human intestine.

### Pathogen Invasion Protection Assay

**FIGURE 11:**
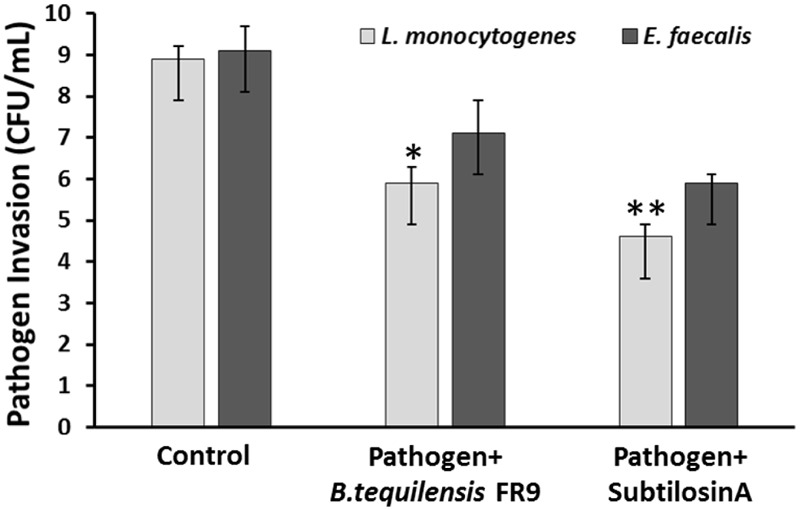
**Invasion protection analysis of pathogen into HCT 116 colon carcinoma cell line with *B. tequilensis* FR9 or purified Subtilosin A.** Results are expressed as mean ± SD (*n* = 3). ^∗^*P* ≤ 0.05, ^∗∗^*P* ≤ 0.01.

Probiotics should adhere to the intestinal epithelial cells and eliminate the adhesion of intestinal pathogens via competitive binding (Das et al., 2013). Inhibition of pathogen invasion by *B. tequilensis* FR9 was demonstrated using HCT-116 colon carcinoma cells. Invasion of *L. monocytogenes* and *E. faecalis* decreased with the addition of either *B. tequilensis* FR9 or purified Subtilosin A. *B. tequilensis* FR9 demonstrated increased invasion protection against *L. monocytogenes* MTCC 657 (5.83 CFU/mL) compared to *E. faecalis* MTCC 439 (7.12 CFU/mL; **Figure 11**). We hypothesize that the increased activity against *L. monocytogenes* might be due to the production of antilisterial protein (Subtilosin A), hence we have used the purified Subtilosin A instead of whole bacterial cell. Results showed increased protection against *L. monocytogenes* (4.63 CFU/mL), which confirms that the Subtilosin A plays a major role in pathogen invasion protection. Similar invasion protection by competitive exclusion as well as antimicrobial production has previously been recorded in many *Bacillus* and *Lactobacillus* spp. (Golowczyc et al., 2011; Das et al., 2013; Khan and Kang, 2016).

## Conclusion

In conclusion, the present study has revealed that *L. gasseri* FR4, *B. tequilensis* FR9, and *L. animalis* FR12 strains isolated from healthy free-range chicken’s GIT displayed better performance in all *in vitro* assays, which is essential to be considered as probiotics. Among them, *B. tequilensis* FR9 was recognized as a reliable probiotic candidate, since they produce antilisterial Subtilosin A and inhibit the invasion of *L. monocytogenes* in human colon cells. Greater *in vitro* adhesion of *B. tequilensis* FR9 to HCT 116 exemplify its higher retention time and decreased adhesion of pathogen to gut which in turn enhances functional efficacy. The production of bacteriocins together with sporulation capacity supports *Bacillus* species with fortified supremacy with respect to their survival in diverse niches. For the first time, we have demonstrated the Subtilosin A production by *B. tequilensis* and have elucidated their probiotic properties. *B. tequilensis* FR9 could be used as a novel probiotic adjuvant for both human and animal foods.

## Author Contributions

ADR, RPR, and MA contributed with the conception and experimental design. RPR, RD, and SH carried out all experiments, MA performed cell line studies and RP-HPLC analysis. MA and RPR analyze and interpret the results. RPR write the manuscript and corrected by ADR and MA. All authors performed a critical revision of the manuscript and approved the final version.

## Conflict of Interest Statement

The authors declare that the research was conducted in the absence of any commercial or financial relationships that could be construed as a potential conflict of interest.
