# Supplementary material for: Purification of Antilisterial Peptide (Subtilosin A) from Novel Bacillus tequilensis FR9 and Demonstrate Their Pathogen Invasion Protection Ability Using Human Carcinoma Cell Line
Source: Front Microbiol. 2016 Dec 1;7:1910. doi: 10.3389/fmicb.2016.01910 (PMC5133052; doi:10.3389/fmicb.2016.01910)
Supplement: Supplementary file 1 [file Data_Sheet_1.pdf]

Purification of antilisterial peptide (SubtilosinA) from novel *Bacillus tequilensis* FR9 and demonstrate their pathogen invasion protection ability using human carcinoma cell line

Rizwana Parveen Rani[a], Marimuthu Anandharaj[b], Subramani Hema[a], Ramasamy Deepika[a] and Abraham David Ravindran[a]*

[a]Department of Biology, The Gandhigram Rural Institute-Deemed University, Gandhigram-624302, Tamilnadu, India.

[b]Biodiversity Research Center, Academia Sinica, Taipei-115, Taiwan.

E-Mail: rizm_green@yahoo.com, anandharaj49@gmail.com, shemagomathi92@gmail.com, deepika.94micro@gmail.com and david_gribiology@rediffmail.com

Table S1: Bacterial strains used in this study to perform antimicrobial activity and their culture conditions.

Genus and Species name	Strain name	Culture conditions
Escherichia coli	MTCC[a]2622	37°C, aerobic, LB[b]
Pseudomonas aeruginosa	MTCC 741	37°C, aerobic, NB[c]
Staphylococcus aureus	MTCC 3160	37°C, aerobic, NB
Klebsiella pneumonia	MTCC 7028	30±2°C, aerobic, NB
Bacillus cereus	MTCC 7278	37°C, aerobic, NB
Listeria monocytogenes	MTCC 657	37°C, aerobic, NB
Enterococcus faecalis	MTCC 439	37°C, aerobic, NB
Candida albicans	MTCC 3017	25°C, aerobic, YEPD[d]
Candida tropicalis	MTCC 184	25°C, aerobic, YEPD[d]
Lactobacillus oris	HMI68	37°C, anaerobic, MRS[e] broth

[a]MTCC: Microbial type culture collection (Chandigarh, India)

[b]LB: Luria broth

[c]NB: Nutrient broth

[d]YEPD: Yeast extract, peptone, dextrose broth

[e]MRS: de Man Rogosa and Sharpe broth

Table S2: Morphological and cultural characteristics of bacterial isolates from *Gallus gallus domesticus*

Bacterial isolates	Physiological characteristics									
	Colony shape	Edge	Elevation	Surface	Pigmentation	Gram staining	Shape	Motility	Curdling property/ odour[a]	Bile tolerance[b] (log CFU/mL)	Acid tolerance[c] (log CFU/mL)
FR1	Irregular	Undulate	Raised	Smooth	No	Positive	Short rods	Non motile	+/ odourless	1.49	2.41
FR2	Irregular	Lobate	Raised	Wrinkled	No	Negative	Short rods	Non motile	- / odourless	1.06	2.08
FR3	Irregular	Entire	Flat	Wrinkled	No	Negative	Cocci	Non motile	+/ odourless	0.48	1.36
FR4	Circular	Entire	Raised	Smooth	No	Positive	Rod	Non motile	++/ odourless	4.34	5.27
FR5	Circular	Lobate	Raised	Smooth	No	Positive	Short rods	Non motile	+/ odourless	1.65	1.81
FR6	Circular	Entire	Flat	Positive	No	Negative	Cocci	Non motile	- / odourless	0.86	1.54
FR7	Irregular	Lobate	Raised	Smooth	No	Positive	Rods	Non motile	+ / odourless	1.93	2.86
FR8	Irregular	Undulate	Raised	Wrinkled	No	Negative	Cocci	Non motile	+ / odourless	0.64	1.99
FR9	Circular	Lobate	Raised	Smooth	Yellow	Positive	Rods	Non motile	++/ Perfect odour	5.86	5.49
FR10	Irregular	Undulate	Flat	Smooth	No	Negative	Rods	Non motile	- / odourless	2.19	2.81
FR11	Circular	Undulate	Raised	Smooth	No	Negative	Cocci	Non motile	+ / odourless	1.03	1.76
FR12	Circular	Entire	Raised	Smooth	No	Positive	Rods	Non motile	++/ odourless	4.94	6.21

[a]**Curdling property:** - negative, + good, ++ very good

[b]**Bile tolerance:** Bacterial cells were grown in MRS broth supplemented with 0.5% bile salt. One mL of sample was collected after 3 h and plated on MRS agar.

[c]**Acid tolerance:** Bacterial cells were grown in MRS broth (adjusted to pH 3.0). One mL of sample was collected after 3 h and plated on MRS agar.

Table S3: Biochemical characteristics of selected bacterial isolates

		FR4	FR9	FR12
**Biochemical Tests**	Indole production	–	+	–
	Methyl red test	–	–	–
	Voges Proskauer test	+	+	+
	Citrate utilization test	–	+	+
	Starch hydrolysis test	–	–	–
	Gelatin hydrolysis test	–	+	–
	Triple Sugar Ion test	–	–	–
	Catalase test	–	+	–
**Growth at different pH**	4.4	+	+	+
	9.6	++	++	++
**Growth at different Temperature**	10°C	–	–	–
	15°C	–	+	–
	45°C	+	++	+
**Growth at different NaCl concentration**	2%	++	++	++
	4%	++	++	++
	6.5%	+	+	+
**Carbohydrate fermentation**	Glucose	++	+++	+++
	Fructose	++	+	++
	Sucrose	+	+	+
	Lactose	++	++	++
	Mannitol	–	+	++
	Maltose	+	+++	++
	Arabinose	–	+	–
	Sorbitol	++	+++	++
	Xylose	–	+++	–
	Mannose	–	–	–
	Galactose	+	++	+

(++) Positive, (+) Partially positive, (-) Negative

Table S4: Antibiotic resistance profiles of FR4, FR9 and FR12 strains against commonly used antibacterial compounds.

Bacterial Isolates	Amp[a]	Amo[b]	Cef[c]	Chl[d]	Ery[e]	Kan[f]	Gen[g]	Met[h]	Pen[i]	Tet[j]	Nov[k]
***L.gasseri* FR4**	+	+	++	++	+	-	+	-	++	+	-
***B.tequilensis* FR9**	++	+	++	+	++	-	++	+	++	++	-
***L.animalis* FR12**	+	-	+	-	+	-	-	+	+	+	-

Zone of inhibition was calculated according to the table given by Clinical and Laboratory Standards Institute (CLSI, 2011)

(++) Resistant, (+)Intermediate resistances, (-)Susceptible

[a]Ampicillin (10 µg), [b]Amoxicillin (5 µg), [c]Cefuroxime (30 µg), [d]Chloramphenicol (30 µg), [e]Erythromycin (20 µg), [f]Kanamycin (30 µg), [g]Gentamycin (10 µg), [h]Metronidazole (20 µg),[i]Penicillin (30 µg), [j]Tetracycline (20 µg), [k]Novobiocin (30 µg).

Table S5: Percentage difference of zone of inhibition size between crude and partially purified bacteriocin of *B. tequilensis* FR9

**Bacterial Isolates**	**CFCS**		**PPB**		**% difference in the inhibition zone size**	
	**FR9**	**AU**	**FR9**	**AU**	**FR9**	**AU**
Pseudomonas aeruginosa MTCC 741	13.33±0.13[e]	1416.88	15.75±0.22	2117.47	18.15	49.44
Escherichia coli MTCC 2622	12.02±0.42[e]	1084.80	13.35±0.14	1422.22	11.06	31.10
Klebsiella pneumoniae MTCC 7028	11.40±0.12[b]	939.60	12.49±0.37	1200.00	9.56	27.71
Diameter mean (Gram-negative)	12.25±0.22	1140.62	13.86±0.24	1579.89	12.92	36.08
Bacillus cereus MTCC 7278	9.18 ±0.18[b]	482.72	9.62±0.15	565.44	4.79	17.13
Staphylococcus aureus MTCC 3160	13.20 ±0.31[cd]	1382.40	15.13±0.11	1929.16	14.62	39.55
Listeria monocytogenes MTCC 657	19.00±0.18[cd]	3250	23.00±0.33	4930.00	21.05	51.69
Enterococcus faecalis MTCC 439	7.12±0.24[e]	146.94	7.23±0.41	162.72	15.16	10.73
Diameter mean (Gram-positive)	12.59±0.29	1225.08	18.65±0.33	1934.25	13.90	29.77
Candida albicans MTCC 3017	17.01 ±0.46[f]	2533.40	19.32±0.23	3372.62	13.58	33.12
Candida tropicalis MTCC 184	13.00±0.51[f]	1330	14.67±0.38	1792.08	12.84	34.74
Lactobacillus oris HMI68	6.98.±0.32[a]	127.20	7.73±0.24	154.52	10.74	21.47

[1]Results were obtained by the neutralized cell free culture supernatants (CFCS) and partially purified bacteriocin (PPB) of *B.tequilensis* FR9 and at the stationary growth phase. [2]Results are expressed as mean ± SD; n = 3. [a–f]Means within a column with different lowercase letters are significantly different ($P \leq 0.05$). NS: not significant ($P>0.05$).

Supplementary figures

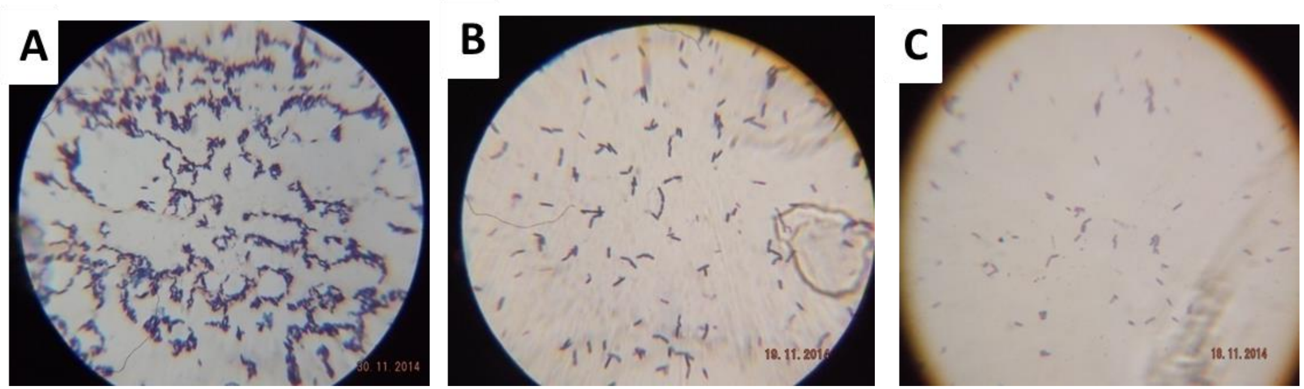

Figure S1: Light microscopic view of isolates FR4 (A), FR9 (B) and FR12 (C).

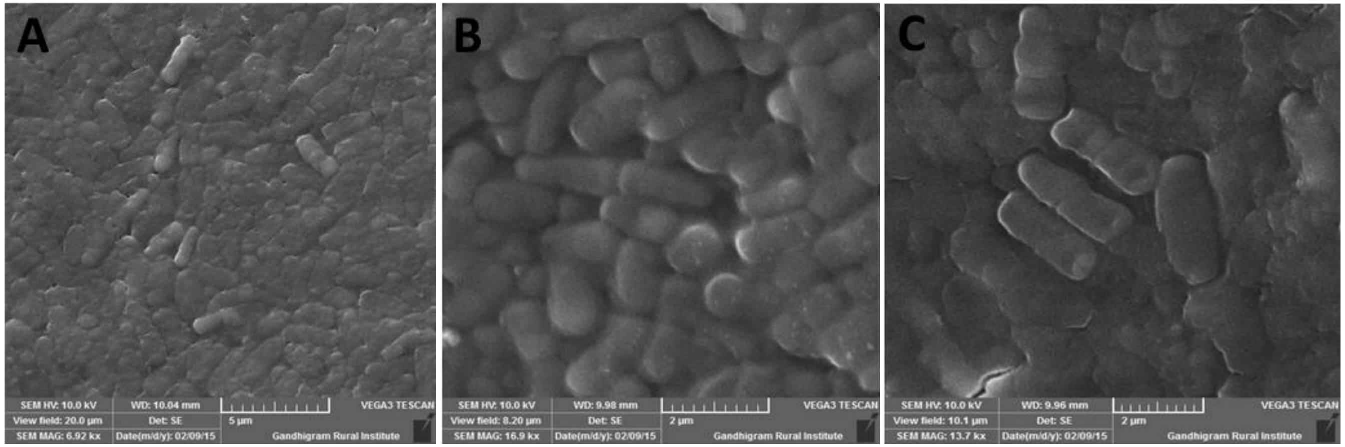

Figure S2: Scanning electron microscopic view of isolates FR4 (A), FR9 (B) and FR12 (C).

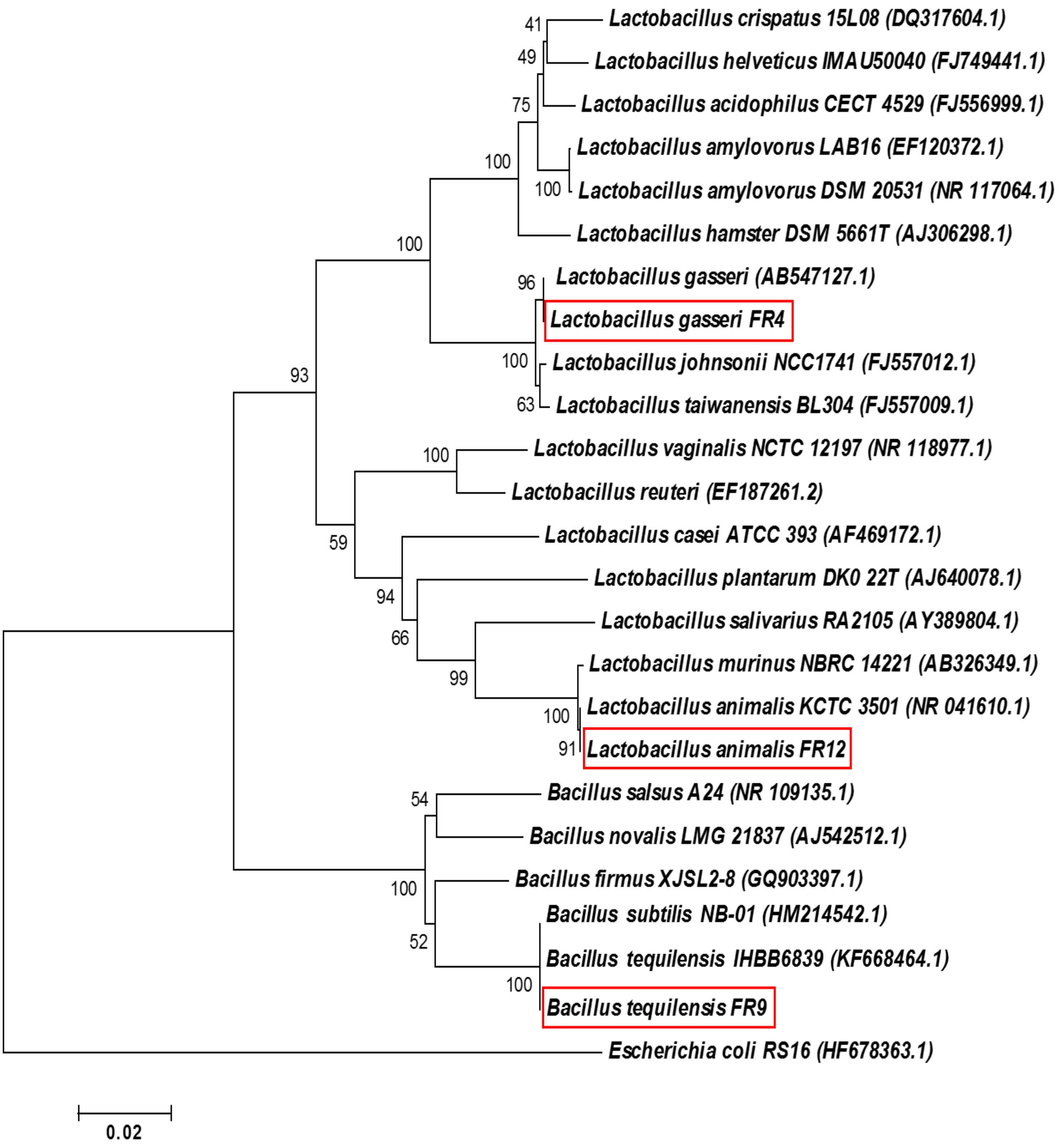

Figure S3: A phylogenetic tree of closely related 16S rRNA gene sequences using Neighbour-joining method. Numbers at branching nodes are bootstrap percentages (based on 1000 replications).
